# A randomized trial of nurse‐administered behavioral interventions to manage anticipatory nausea and vomiting in chemotherapy

**DOI:** 10.1002/cam4.2863

**Published:** 2020-01-19

**Authors:** Jonathan J. Hunter, Robert G. Maunder, Dawen Sui, Mary Jane Esplen, Alejandro Chaoul, Michael J. Fisch, Roland L. Bassett, Marlys M. Harden‐Harrison, Lore Lagrone, Lucas Wong, Luis Baez‐Diaz, Lorenzo Cohen

**Affiliations:** ^1^ Sinai Health System The University of Toronto Toronto ON Canada; ^2^ Department of Biostatistics The University of Texas MD Anderson Cancer Center Houston TX USA; ^3^ Princess Margaret Cancer Centre de Souza Institute Toronto ON Canada; ^4^ Department of Palliative, Rehabilitation, and Integrative Medicine The University of Texas MD Anderson Cancer Center Houston TX USA; ^5^ Department of General Oncology The University of Texas MD Anderson Cancer Center Houston TX USA; ^6^ Baylor Scott and White Health Temple TX USA; ^7^ Puerto Rico MUNCORP San Juan PR USA

**Keywords:** classical conditioning/music therapy/relaxation therapy, nausea/vomiting/anticipatory nausea

## Abstract

**Purpose:**

Chemotherapy side effects diminish quality of life and can lead to treatment delay. Nausea and vomiting can occur prior to chemotherapy because of classical conditioning. We studied the effects of 20‐minute behavioral interventions, administered by oncology nurses, of higher intensity (mindfulness relaxation—MR) or lower intensity (relaxing music—RM), on anticipatory nausea and vomiting (ANV).

**Patients and methods:**

Patients undergoing chemotherapy for solid tumors were randomized to MR (N = 160), RM (N = 159), or standard care SC (N = 155). Subjects were mostly female (91.8%) and white (86.1%) with breast cancer (85%). Most patients had early stage disease (Stage I: 26%; II: 52.9%; III: 19%; IV: 0.1%). Anticipatory nausea and vomiting were assessed at the midpoint and end of the chemotherapy course using the Morrow Assessment of Nausea and Emesis (MANE).

**Results:**

Compared to SC, there was reduced anticipatory nausea at the midpoint of chemotherapy in those receiving MR (OR 0.44, 95% CI 0.20‐0.93) and RM (OR 0.40, 95% CI 0.20‐0.93), controlling for age, sex, cancer stage, and emetogenic level of chemotherapy. There was no difference between treatment groups in anticipatory nausea at the end of chemotherapy or in anticipatory vomiting and postchemotherapy nausea and vomiting at either time point.

**Conclusion:**

A brief nurse‐delivered behavioral intervention can reduce midpoint ANV associated with chemotherapy.

## Graphical abstract

Chemotherapy administration can result in conditioned nausea and vomiting. This multi‐centred randomized controlled trial demonstrates the superiority of 2 preemptive behavioural interventions in reducing conditioned nausea.

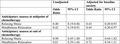

## 1 INTRODUCTION

Cancer chemotherapy has side effects, such as alopecia, nausea, vomiting, anorexia, stomatitis, diarrhea, and fatigue^1^ which are the direct result of the cytotoxicity of the chemotherapy agents, and which will affect as many as 86% of chemotherapy patients.^1^ In a course of repeated exposures to chemotherapy, some of these side effects may also occur *prior* to the administration of chemotherapy agents.^2^, ^3^, ^4^ In particular, anticipatory nausea and vomiting (ANV) is common, occurring in 10% to 30% of patients undergoing chemotherapy.^5^, ^6^, ^7^

The single most powerful predictor of ANV is the experience of nausea and vomiting *after* chemotherapy.^8^ Thus, ANV is most commonly understood to be a classically conditioned response.^4^ Much as Pavlov trained dogs to salivate upon hearing a bell that had been paired with food, the normal chemotherapy regimen "teaches" patients to pair nausea and/or vomiting with chemotherapy‐associated cues, such as the smell of a chemotherapy unit and other sights and sounds of the hospital setting. ANV patients typically develop ANV by the third chemotherapy session after which they may find themselves profoundly nauseated or vomiting up to 48 hours prior to chemotherapy,^9^ or whenever presented with cues that are reminiscent of treatment. Symptoms of ANV usually mount in intensity as patients approach the hospital and can therefore have an impact on treatment compliance or satisfaction. Other factors, such as anxiety and negative expectancies, as well as demographic features (including being female and less than 50 years of age) have been shown to increase susceptibility to ANV.^3^

Nausea and vomiting are less of a problem now for patients undergoing chemotherapy due to improvements in antiemetic medications.^10^, ^11^ This is especially true for acute episodes of vomiting. Nonetheless, conditioned symptoms remain an important complication of chemotherapy because once established, they are refractory to pharmacological treatment.^3^ Although resistant to medication, conditioned symptoms can be responsive to behavioral interventions. Burish et al^12^, ^13^ originally reported decreases in heart rate, blood pressure, and nausea in patients who received progressive muscle relaxation and guided imagery compared to controls. However, when a therapist was not present the effect was obliterated. Morrow et al^14^ showed electromyography biofeedback decreased distress, anxiety, and nausea. They also found that systematic desensitization decreased ANV. Hypnosis and guided imagery practiced throughout chemotherapy can also reduce ANV.^5^, ^15^

Despite the success of behavioral interventions over many years in reducing the intensity of ANV, these strategies are not widely employed. One limitation is the high cost of adding mental health professionals to the oncology team. In a conditioning paradigm, it is also more difficult to extinguish an association that has been established than to prevent its occurrence in the first place.^12^, ^16^ Thus, an alternative approach would be to have oncology nurses deliver a behavioral intervention, and to do it preemptively.^17^ Lerman^18^ has shown that relaxation training delivered by nurses can be effective.^18^ Lerman also noted that efficacy could likely be increased further if nurses were available to deliver "booster" relaxation sessions at the time of subsequent chemotherapy treatments.

The present study tested nurse‐delivered behavioral interventions designed to prevent ANV. A higher intensity intervention, consisting of relaxation and meditation techniques, was compared to a low intensity intervention—listening to relaxing music, and to treatment as usual. Trained oncology nurses delivered the behavioral interventions to patients prior to the first administration of chemotherapy in order to prevent conditioned side effects and patients were directed to use recorded sessions as boosters prior to subsequent treatments. The intervention was embedded within the normal chemotherapy protocol to maximize convenience and potential clinical generalization and in an attempt to increase interpersonal engagement between the patient and the treatment team.

The higher intensity intervention consisted of a script which was adapted from techniques of mindfulness meditation,^19^ guided imagery,^20^, ^21^, ^22^ and yoga practices,^23^, ^24^, ^25^ for medically ill patients. We refer to it as mindfulness relaxation (MR). The lower intensity intervention consisted of listening to relaxing music (RM) for an equivalent duration of time. We tested these interventions in a controlled trial in which patients who received at least four cycles of adjuvant chemotherapy were randomized to one of these interventions or to standard care, which included chemotherapy education (SC).

## 2 HYPOTHESES

The primary hypothesis was that, for cancer patients receiving chemotherapy, MR reduces the occurrence of ANV at the midpoint and endpoint of chemotherapy more effectively than a less intensive intervention or than standard care. Secondary hypotheses were that participants treated with MR would have shorter duration and lower severity of nausea and vomiting compared to those treated with RM and SC.

## 3 METHODS

The study (ClinicalTrials.gov Identifier: NCT00086762) was a randomized, three‐arm trial of MR vs RM vs SC in patients receiving chemotherapy. Eligible subjects were: 1) over 18 years of age; 2) undergoing at least four cycles of cancer chemotherapy treatment for a solid tumor; 3) naïve to previous treatment with chemotherapy; 4) without evidence of metastatic disease; 5) able to communicate in English or Spanish; 6) not experiencing any major psychiatric illness; and 7) had an expected survival time of at least 1 year.

The study was performed through the NCI‐supported Community Clinical Oncology Program (CCOP) through The University of Texas MD Anderson Cancer Center Research Base from 2008 to 2017, which included 5‐year survival for all patients. The MD Anderson CCOP consisted of a network of community‐based oncology practices across the United States that participated in trials of cancer interventions. The Institutional Review Board (IRB) of MD Anderson, the NCI, and that of each community oncology site approved the study. The characteristics of patients is shown in Table 1.

**Table 1:** Characteristics of participants

Characteristic	Mindfulness relaxation N = 160	Relaxing music N = 159	Standard care N = 155	*P*
	N (%)	N (%)	N (%)	
Age (y)				
<60	109 (68)	106 (67)	105 (68)	
≥60	51 (32)	53 (33)	50 (32)	.96
Sex				
Male	14 (9)	13 (8)	12 (8)	
Female	146 (91)	146 (92)	143 (92)	.95
Race and ethnicity				
White non‐Hispanic	110 (69)	106 (67)	102 (66)	
Black non‐Hispanic	16 (10)	17 (11)	21 (14)	
Hispanic	32 (20)	33 (21)	31 (20)	
Asian non‐Hispanic or unknown	1 (1)	3 (2)	1 (1)	.88*
Living with marital or common‐law partner	106 (66)	102 (64)	96 (62)	.86
Employed full‐time	68 (43)	68 (43)	76 (49)	.31
Income >$50 000	61 (38)	58 (37)	60 (39)	.89
Any postsecondary education	102 (66)	100 (67)	104 (71)	.56
Cancer type				
Breast	133 (83)	138 (87)	130 (84)	
Gastrointestinal	11 (7)	9 (6)	10 (7)	
Other	16 (10)	12 (8)	15 (10)	.92
Cancer stage				
I	46 (29)	42 (26)	38 (25)	
II	83 (52)	86 (54)	82 (53)	
III	28 (18)	30 (19)	33 (21)	
IV	3 (2)	1 (1)	2 (1)	.91*
Emetic risk of chemotherapy				
Low (10%‐30%)	5 (3)	3 (2)	3 (2)	
Moderate (30%‐60%)	39 (24)	40 (25)	40 (26)	
Moderate (60%‐90%)	90 (56)	88 (55)	85 (55)	
High (90%‐100%)	26 (16)	28 (18)	27 (17)	.99

* Fisher’s exact test.

The required sample size, using a targeted standard deviation of 0.15, based on previous data, was 133 per group, or n = 400 overall. Patients with newly diagnosed cancer, who were scheduled to undergo chemotherapy, were adaptively randomized^26^ in a ratio of 1:1:1 to MR, RM, or SC. Adaptive randomization is a means of randomizing patients to minimize potential bias between treatment arms.^26^ In this case, we used minimization, and considered the following factors, Cancer Type, Disease Stage, Gender, Age, Emetogenic level of chemotherapy, and CCOP Center.

Subjects completed all self‐report measures at baseline (prior to randomizing), at the midpoint of chemotherapy (defined as session 2 of a 4 course chemotherapy protocol, or session 3 of a 6 course protocol) and at the end of chemotherapy. Patients also completed brief assessments prior to each cycle of chemotherapy. Additionally, data were abstracted from patient charts including age, sex, family cancer history, race, type of cancer, staging information, type of surgical procedure, treatment regimen (eg, chemotherapy, radiation), the day and time of treatments, medical complications, and number of medical visits. Although patients completed a number of psychosocial and quality of life measures, the primary outcome was nausea and vomiting. The current paper presents the data for the primary outcomes.

### 3.1 Nurse training and delivery of mindfulness relaxation

Treatment manuals were developed to standardize the intervention. MR consisted of a single exercise, composed of guided mindfulness, imagery, and relaxation practices, of approximately 20 minutes’ duration which was repeated throughout the course of chemotherapy. Oncology nurses from the CCOP network volunteered to be trained in the intervention by the study team. Following training, each nurse prepared a master recording of the MR exercise to use with his or her patients.

Prior to chemotherapy, during the first meeting with the oncology nurse, a patient was given the standard chemotherapy education, and then taught the MR technique by the nurse in an individual session. The nurse also provided the patient with a copy of the nurse's recording for practice at home, because a recording in the nurse's own voice has previously been shown to promote self‐soothing.^27^ Participants were instructed to practice at home at least once daily throughout chemotherapy treatment. In addition, each time the patient attended a chemotherapy session, he or she used the recording to practice MR. For those subjects who had Spanish as a first language (n = 96) the intervention was conducted in Spanish, utilizing a translation of the MR script into Spanish, and then back into English to assure its accuracy. Although there was an intention to perform follow‐up calls to remind subjects to practice, this became unfeasible due to competing time demands for staff, and was not carried out.

### 3.2 Delivery of relaxing music

The RM group received a recording to be utilized in a manner identical to the MR, but which did not contain any specific instructions on relaxation or meditation; rather it consisted of relaxing music with nature sounds or a vocal track. They also received general information on the management of symptoms related to chemotherapy in a session of equivalent time to the MR training session.

### 3.3 Delivery of standard care

In the SC group, patients received general information on the management of symptoms related to chemotherapy as would be typical of that CCOP site. The same duration of individual contact with the nurse occurred in all conditions.

### 3.4 Instruments

Nausea and vomiting were the primary outcomes of the trial and were measured with the Morrow Assessment of Nausea and Emesis (MANE)^28^ at the final infusion and also at the ‘midpoint’, defined as session 2 of a 4 course chemotherapy protocol, or session 3 of a 6 course protocol. The MANE probes the incidence, severity, and duration of both anticipatory and postchemotherapy nausea and vomiting.^28^ It was validated on 500 consecutive oncology outpatients with various tumors and treatment regimens. The test‐retest reliability for different components of nausea and vomiting ranged from 0.76 to 0.96. Measures of convergent and divergent validity also demonstrated good support for the construct validity of the measure.^28^ The emetogenic level of the chemotherapy was derived from the Hesketh scale,^29^ a widely recognized standard for evaluating this characteristic of chemotherapy agents.

Immune measures were also obtained on a subset of patients, but are not yet analyzed so will not be included here. Results other than nausea and vomiting and quality of life will be reported subsequently.

### 3.5 Analyses

Measures of nausea and vomiting were only assessed at midpoint and end of treatment. As such, analyses included all participants who completed the MANE within the cycle being analyzed. Rates of nausea and vomiting and the severity of nausea in each treatment group were calculated. The occurrence of anticipatory nausea (any/none) prior to the midpoint of chemotherapy was compared between treatment groups using binary logistic regression, with age, sex, cancer stage, and emetogenic level of chemotherapy agent(s) entered as covariates. Nausea before the midpoint of treatment (any/none) was the outcome variable and treatment group (MR, RM, SC) was the independent variable. This analysis was repeated for the occurrence of anticipatory nausea (any/none) prior to the endpoint of chemotherapy. Severity of nausea before the midpoint of chemotherapy (rated as none, very mild/mild, moderate/severe) was compared between treatment groups by chi‐square test. Participants who were excluded from analyses based on missing data were compared to those who were included with respect to age, sex, cancer stage, and emetogenic level of the chemotherapy agent(s). The effect of intervention on quality of life (total FACT score, and each subscale) over all measurement time points was tested with General Linear Model repeated measures analysis, controlling for baseline state anxiety. All tests were two‐sided with significance set at *P* < .05.

## 4 RESULTS

**Figure 1:**
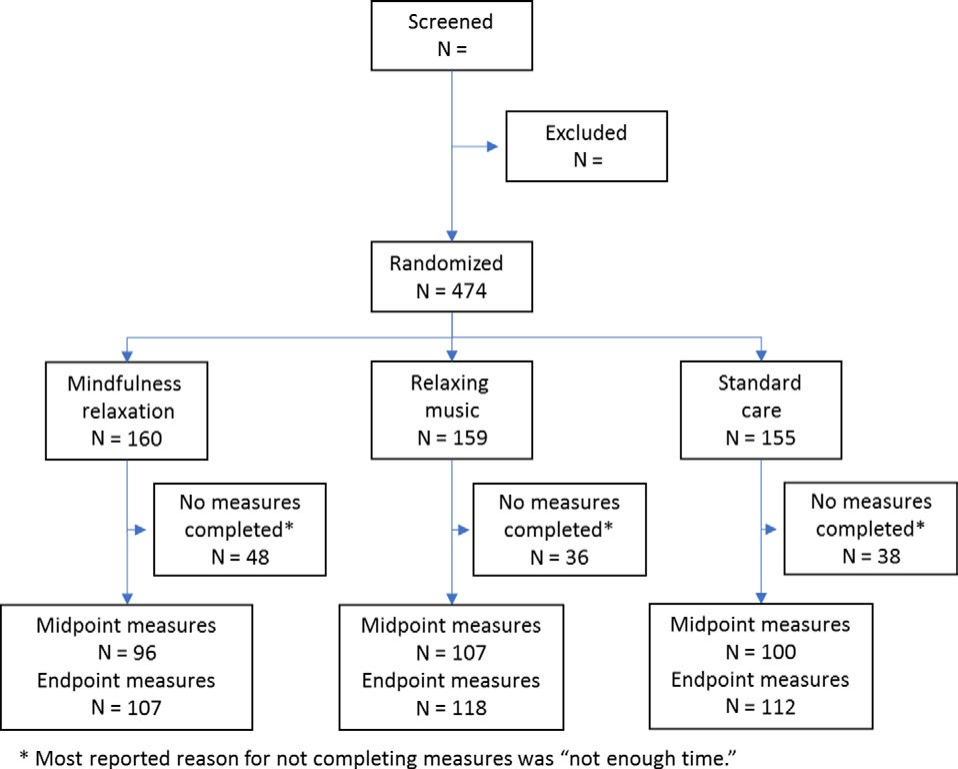
Subject allocations to treatment arms

We are reporting on the primary outcome alone, as survival analyses have not yet been completed. In total, 474 patients participated. (See Figure 1 for subject allocations). Subjects were recruited through 12 different CCOP sites in seven states and Puerto Rico. Sites varied in numbers recruited with the preponderance of subjects coming from centers in Texas, Michigan (two sites), and Puerto Rico. Participants were treated for breast cancer (85%), gastrointestinal cancer (6%) and other malignancies (9%). The prototypic participant was white (86.1%) and female (91.8%). Participants were assigned to MR (N = 160), RM (N = 159) or SC (N = 155). Further characteristics of participants and their distribution across intervention groups are described in Table 1. The groups differed with respect to state anxiety at baseline, MR 44.5 ± 12.7, RM 40.3 ± 12.5and SC 42.0 ± 12.7 (*P* = .017, Fisher's exact test). There were no significant differences amongst groups on any of the other variables reported in Table 1. One hundred and twenty‐two (25.7%) patients only completed baseline measures and were excluded from this analysis as the primary outcome (MANE) was only assessed in the middle and end of chemotherapy. There were a similar number of drop‐outs in each of the three groups. Excluded patients did not differ from those included based on age, sex, cancer stage, or emetogenic level of the chemotherapy agent(s) (statistics not shown).

**Table 2:** Nausea and vomiting during the previous chemotherapy session and before the current one, as measured at the midpoint and endpoint of chemotherapy

	Midpoint of chemotherapy			Endpoint of chemotherapy		
	Mindfulness relaxation	Relaxing music	Standard care	Mindfulness relaxation	Relaxing music	Standard care
	N = 96	N = 107	N = 100	N = 112	N = 122	N = 117
Nausea during previous chemo (Chemotherapy‐induced nausea)						
None	64 (66.7%)	70 (65.4%)	59 (59.0%)	60 (53.6%)	66 (54.1%)	51 (43.4%)
Any‐very mild or mild	12 (12.5%)	16 (15.0%)	15 (15.0%)	24 (21.4%)	16 (13.1%)	32 (27.4%)
Any‐moderate or worse	18 (18.8%)	19 (17.8%)	26 (26.0%)	28 (25.0%)	39 (32.0%)	33 (28.2%)
Missing	2 (2.1%)	2 (1.9%)	0 (0.0%)	0 (0.0%)	1 (0.8%)	1 (0.8%)
Vomiting during previous chemo (Chemotherapy‐induced vomiting)						
None	80 (83.3%)	93 (86.9%)	82 (82.0%)	95 (84.8%)	102 (83.6%)	89 (76.1%)
Any	13 (13.5%)	12 (11.2%)	15 (15.0%)	9 (8.0%)	13 (10.7%)	22 (18.8%)
Missing	3 (3.1%)	2 (1.9%)	3 (3.0%)	8 (7.1%)	4 (3.3%)	6 (5.1%)
Nausea before current chemo (Conditioned/anticipatory nausea)						
None	83 (86.5%)	94 (87.9%)	72 (72.0%)	87 (77.6%)	93 (76.2%)	88 (75.2%)
Any‐very mild or mild	10 (10.4%)	10 (9.3%)	14 (14.0%)	13 (11.6%)	12 (9.8%)	15 (12.8%)
Any‐moderate or worse	1 (1.0%)	2 (1.9%)	9 (9.0%)	3 (2.7%)	7 (5.7%)	5 (4.3%)
Missing	2 (2.1%)	1 (0.9%)	5 (5.0%)	9 (8.0%)	8 (6.6%)	9 (7.7%)
Vomiting before current chemo (Conditioned/anticipatory vomiting)						
None	89 (92.7%)	103 (96.3%)	92 (92.0%)	97 (86.6%)	106 (86.9%)	104 (88.8%)
Any	3 (3.1%)	1 (0.9%)	5 (5.0%)	3 (2.7%)	5 (4.1%)	4 (3.4%)
Missing	2 (2.1%)	3 (2.8%)	3 (3.0%)	12 (10.7%)	11 (9.0%)	9 (7.7%)

Table 2 shows the occurrence and severity of nausea and vomiting pre‐ and postchemotherapy at the midpoint and endpoint of chemotherapy treatment in each of the three intervention groups. At the midpoint of chemotherapy treatment, participants who received MR or RM experienced nausea before chemotherapy significantly less often than participants who received SC (Table 3), indeed, about half as often (Table 2). Of those who experienced prechemotherapy nausea at the midpoint of chemotherapy, it was of moderate to intolerable severity in 1 (1.0%), 2 (1.9%), and 9 (9.0%) participants who received MR, RM, and SC respectively. The difference between treatment groups in the severity of nausea at the midpoint (none, very mild/mild, moderate/severe, Table 3) was significant (Chi‐square = 12.7, *P* =.01), and remained so after controlling for baseline anxiety. The prevalence of vomiting was low and the difference between treatment groups was not significant. There was no significant difference in the prevalence of anticipatory nausea between groups at the endpoint of therapy (Table 3) nor in postchemotherapy nausea and vomiting at either time point. There was no difference between intervention groups in the trajectory of quality of life (statistics not shown).

**Table 3:** Nausea before the previous chemotherapy session, as measured at the midpoint and endpoint of chemotherapy

	Unadjusted		Adjusted for baseline anxiety	
	Odds Ratio*	95% CI	Odds Ratio*	95% CI
Anticipatory nausea at midpoint of chemotherapy				
Relaxing music	0.40	0.19‐0.86	0.43	0.20‐0.93
Mindfulness relaxation	0.42	0.19‐0.91	0.44	0.20‐0.97
Anticipatory nausea at end of chemotherapy				
Relaxing music	0.90	0.45‐1.80	0.89	0.44‐1.82
Mindfulness relaxation	0.81	0.39‐1.66	0.72	0.34‐1.51

* Binary logistic regression with standard care as reference group.

## 5 DISCUSSION

This study demonstrated that two behavioral interventions reduced the incidence of mid‐chemotherapy ANV in patients receiving chemotherapy for cancer. However, there was no difference in incidence or severity of vomiting. This is due to the fact that vomiting was well controlled in all patients, and is likely a testament to the advances made in recent years in antiemetic medication strategies.^10^, ^11^ These behavioral interventions were provided by available staff, with little additional time required beyond treatment as usual. This study was implemented in 13 cancer treatment centers, in 2 languages, with 474 patients, suggesting that widespread implementation to reduce patient burden in chemotherapy treatment is feasible.

Notably, there was no demonstrated difference in outcome between the more active behavioral intervention (MR) and the less active intervention (RM), both of which were superior to usual care (TAU). Therefore, the greater complexity associated with meditation‐like programs such as MR may not result in better outcomes compared to simply listening to relaxing music. This suggests that behavioral intervention is effective, but need not be intensive. However, in the current study, both active interventions were applied for a short period of time. Longer term meditation practice has been associated with other benefits including neurogenesis,^30^ which may not result from brief interventions such as MR. Although the study design does not allow us to distinguish between the effects of staff taking the time to address a patient's need for self‐soothing and the direct effects of engaging in MR or RM, we expect that the former is a significant active component of these behavioral interventions, along with the interventions activating the relaxation response, resulting in a dampening of sympathetic nervous system activation.^31^ It is also relevant that previous studies have found that listening to music is an effective intervention in some circumstances, including anxiety associated with chemotherapy.^32^, ^33^ Identifying the specific components of MR and RM that reduce nausea requires further research.

This study found no significant group difference in ANV at the endpoint of treatment, suggesting that the effect of the interventions was significant but not sustained. Previous mind‐body research has shown a dose‐response between frequency of practice and outcomes. Although the original design included the subjects keeping diaries to tabulate frequency and duration of intervention use, this was experienced as burdensome and inconsistently performed, meaning that data collection for frequency of practice (or ‘dose’ of intervention) was unreliable and prevented testing of the relationship between consistency of practice and outcome. Therefore, we cannot state if variations in practice account for these results, but lack of a sustained effect throughout chemotherapy may be due to the lack of practice of the intervention over time or inconsistent implementation, after initial training, at subsequent cycles of chemotherapy. Therefore, we cannot state if variations in practice account for the lack of a sustained effect throughout chemotherapy, although it is plausible. Conceivably, the behavioral practice could also avoid or reduce side effects associated with the antiemetic regimens as well.^34^

There are a number of limitations to this trial. As with many behavioral studies in clinical settings, recruitment was a challenge. This was especially so for this trial as we were recruiting from smaller centers with frequent turnover of staff, at sites more familiar with pharmacological studies than behavioral interventions. In addition, each site had multiple studies to which they recruited patients, so overlap in inclusion criteria was an additional factor leading to slow accrual. There was a sizable number of patients who completed baseline, were randomized, and did not complete any follow‐up assessments. These patients were not included in the analyses, as doing so would require too many assumptions. However, there was a similar number of drop‐outs between groups and there were no differences between those with baseline only versus those with follow‐up data on any variable assessed. This suggests that even assigning everyone the worst score would not change the outcomes. As noted, frequency of practice in the behavioral interventions was not consistently assessed, therefore it was not possible to examine any association between exposure to the practice and outcomes. Frequency and type of antiemetics used in this study was also unavailable due to the lack of adherence to daily diary data recording. Therefore we cannot comment on the possibility that some subjects received either significantly more or less effective protocols, which could change this finding. However, all sites followed the basic NCCN guidelines in the prophylactic and ongoing use of antiemetics and each site randomized patients equally across the three groups. Similarly, although there were quality control checks for the training of the nurses and initial delivery of the interventions, ongoing fidelity checks were not conducted. This could have resulted in inconsistent delivery of the interventions. As the nurses were trained in delivery of research protocols consistency was likely, but not assured. The study was also not conducted in a blinded manner as to the two behavioral intervention groups. Future behavioral research should try and make every effort to conduct the trial in a blinded manner, at least in regard to the specific behavioral interventions when more than one is included, and to thoroughly assess engagement and adherence.

In summary, the current trial found that easy to delivery behavioral interventions, taught to patients by an existing staff member at the start of chemotherapy, resulted in reduction in ANV at the midpoint of the course of chemotherapy. The overall effect of the behavioral programs was relatively modest, but it remains encouraging that mid‐treatment incidence of ANV was reduced with such low‐burden behavioral interventions.

## CONFLICT OF INTEREST

Dr Fisch is employed by Aim Specialty Health, a subsidiary of Anthem, Inc All other authors declare no conflicts of interest.

## AUTHOR CONTRIBUTIONS

Jonathan Hunter, Lorenzo Cohen, Mary Jane Esplen, Alejandro Chaoul, and Michael Fisch were involved in conception and design. Lorenzo Cohen and Michael Fisch were involved in financial support. Lore Lagrone and Marlys Harden‐Harrison were involved in administrative support. Lucas Wong and Luis Baez‐Diaz were involved in provision of study material or patients. Lore Lagrone, Marlys Harden‐Harrison, and Dawen Sui were involved in collection and assembly of data. Jonathan Hunter, Lorenzo Cohen, Robert Maunder, Roland Bassett, and Dawen Sui were involved in data analysis and interpretation. All authors were involved in manuscript writing, final approval of manuscript, and accountable for all aspects of the work.

## Data Availability

The data that support the findings of this study are available on request from the corresponding author. The data are not publicly available due to privacy or ethical restrictions.
